# Strategic Directions in Preventive Intervention Research to Advance Health Equity

**DOI:** 10.1007/s11121-022-01462-5

**Published:** 2022-12-05

**Authors:** Rhonda C. Boyd, Felipe González Castro, Nadine Finigan-Carr, Scott K. Okamoto, Allison Barlow, Bo-Kyung Elizabeth Kim, Sharon Lambert, Jacqueline Lloyd, Xinzhi Zhang, Crystal L. Barksdale, Daniel M. Crowley, Mildred Maldonado-Molina, Ezemenari M. Obasi, Anne Kenney

**Affiliations:** 1 Children’s Hospital of Philadelphia, Philadelphia, PA USA; 2 Arizona State University, Phoenix, AZ USA; 3 University of Maryland Baltimore, Baltimore, MD USA; 4 University of Hawaii Cancer Center, Honolulu, HI USA; 5 John Hopkins Center for American Indian Health, Baltimore, MD USA; 6 University of Southern California, Los Angeles, CA USA; 7 George Washington University, Washington, DC USA; 8 Office of Disease Prevention, National Institutes of Health, Rockville, MD USA; 9 National Center for Advancing Translational Sciences, National Institutes of Health, Bethesda, MD USA; 10 National Institute of Mental Health, National Institutes of Health, Bethesda, MD USA; 11 Penn State University, State College, PA USA; 12 University of Florida College of Medicine, Gainesville, FL USA; 13 University of Houston, Houston, TX USA

**Keywords:** Ecosystemic framework, Health equity statement, Health inequities, Health equity, Health disparities, Evidence-based preventive interventions, Intersectionality, Cultural factors

## Abstract

As commissioned by the Society for Prevention Research, this paper describes and illustrates strategic approaches for reducing health inequities and advancing health equity when adopting an equity-focused approach for applying prevention science evidence-based theory, methodologies, and practices. We introduce an ecosystemic framework as a guide for analyzing, designing, and planning innovative equity-focused evidence-based preventive interventions designed to attain intended health equity outcomes. To advance this process, we introduce a health equity statement for conducting integrative analyses of ecosystemic framework pathways, by describing the role of social determinants, mechanisms, and interventions as factors directly linked to specific health equity outcomes. As background, we present health equity constructs, theories, and research evidence which can inform the design and development of equity-focused intervention approaches. We also describe multi-level interventions that when coordinated can produce synergistic intervention effects across macro, meso, and micro ecological levels. Under this approach, we encourage prevention and implementation scientists to apply and extend these strategic directions in future research to increase our evidence-based knowledge and theory building. A general goal is to apply prevention science knowledge to design, widely disseminate, and implement culturally grounded interventions that incrementally attain specific HE outcomes and an intended HE goal. We conclude with recommendations for conducting equity-focused prevention science research, interventions, and training.

## Strategic Directions in Preventive Intervention Research to Promote Health Equity

*Health disparities* (HDs) and *health inequities* (HIs) have existed for several decades in the USA as pervasive public health problems (Koh et al., 2011; Obasogie et al., 2017). Structural inequities are produced and maintained by complex interactions among social determinants of health that include historical events, unjust social policies, barriers to health care access, discriminatory practices, and underlying systems of racism and prejudice. To advance *health equity* (HE), the USA must acknowledge our nation’s unjust history of racism and bias and rectify unjust actions that have created and sustained HIs. This reality calls for concerted efforts to counter inequities by developing equity-focused interventions implemented through specific ecological pathways to attain HE outcomes and goals.

COVID-19 as well as recent civil unrest and current economic conditions have brought increased attention to inequities. As a result, major scientific societies have advocated and taken actions to advance HE in research and practice (Brownson et al., 2021; Shelton & Adsul, 2021; Volpe et al., 2019). While these efforts are important, clearly HE cannot be achieved without changing the social structures, systems, and ideologies that historically have produced and maintained inequities.

### Society for Prevention Research Disparities-Equity Task Force

In 2017, the Society for Prevention Research commissioned the Disparities-Equity Task Force to examine “the role of prevention science in reducing HDs and increasing HE.” This task force consisted of established prevention scientists from across the USA with expertise in HD research and experience in working with diverse populations at risk for HDs. Task force members represented diverse rural, urban, ethnocultural, and/or lower SES communities. This task force was charged with summarizing current prevention science evidence and identifying future directions for preventive intervention research to advance equitable health and well-being for all, with particular attention to oppressed groups and communities.

As defined years ago, prevention science utilizes evidence-based approaches to mitigate or remedy problems before they occur by identifying and targeting precursors and unfair and unjust structural conditions that erode health and well-being (Coie et al., 1993). It is time now to apply the robust evidence accrued in prevention science over the past 50 years to develop and translate interventions that can resolve unfair and unjust structural conditions to attain HE goals.

This paper was commissioned by the Board of Directors of the Society for Prevention Research. It provides a guiding framework for prevention scientists to develop research initiatives that purposefully target the promotion of HE, with exemplary methods, interventions, and the application of new innovations in the field. We conclude with specific recommendations on how prevention science can strategically advance research, practice, and policy towards achieving HE outcomes and goals. This paper serves as a charge to the field of prevention science to utilize a HE lens in the design of future evidence-based preventive interventions (EBPIs).

## Concepts, Constructs, Theories, and Models for Equity-Focused Prevention Science

As a point of departure, it is critical to establish a collective understanding of HDs, HIs, and HE as social justice constructs (see Table 1). Definitions constitute a starting point; they alone are not sufficient to fully understand these social justice constructs and their application within the ecological contexts of real-world situations. Accordingly, in Table 1, we include a column on contextual issues and nuances to consider in the real-world application of these important constructs.

**Table 1:** Definitions and contexts for select social justice constructs

Construct	Definition	Contextual Issues and Nuances
• **Intersectionality**	• Intersectionality is a framework, theory, and approach to social justice that is applicable to several topic domains that include prejudicial attitudes, experiences of discrimination, friendship, resilience, and activism (Parent et al., 2013) • “Intersectionality describes how social, economic, and historical context intersects with multiple forms of inquiry and identity (e.g. the interconnectedness of race, class, gender…” (Volpe et al., 2019, p. 308)	• In virtually every society across the world social advantage and disadvantage are influenced by the *intersectionality* of socioeconomic, gender, ethnic, age, and other identity-related factors (Braveman, 2006, p. 170) • *Intersectionality* can be used, “to understand systemic-level ways in which interlocking social identity positions may change the specific target for racism interventions to reduce racial health disparities.” (Volpe et al., 2019, p 311) • “When applied, intersectionality calls researchers to attend to both structural and individual-level variables, such as exploring ways that interlocking forms of oppression simultaneously influence a person’s life experiences.” (Volpe et al., 2019, p. 308)
• **Health Disparities **	• A health disparity refers to, “… a difference in which disadvantaged social groups -such as the poor, racial/ethnic minorities, women, or other groups who have persistently experienced social disadvantage or discrimination – systematically experience worse health or greater health risks than more advantaged groups.” (Braveman, 2006, p. 167)	• These differences contrast a disadvantaged group with an advantaged group (Braveman, 2006) • These differences are: unjust, potentially avoidable, and shaped by policies that adversely affect poor, racial/ethnic, and other discriminated groups, compromising their health and well-being (Braveman, 2006)
• **Health Inequities **	• Health inequities (HIs) are differences in death and disease that have been imposed systemically by “unequal access to social or economic resources” (Kneipp et al., 2018, p. 233) •Health inequities constitute “disparate health outcomes [that are] often produced by particular social, economic, political, and legal arrangements rather than simply being a natural part of the social world.” (Obasogie et al., 2017, p. 326)	• Health inequities are created by unjust structural conditions that unfairly benefit a privileged social group while imposing multiple oppressive conditions and barriers on one or more minoritized and thus marginalized groups (Brownson et al., 2021; Shelton & Adsul, 2021; Trinh-Shevrin et al., 2015) • Existing inequities operate as obstacles that must be removed to achieve equity and health equity. (Minkler et al., 2019)
• **Health Equity **	• Health equity “implies [that] everyone should have a fair opportunity to attain their fill health potential, and that no one should be disadvantaged from achieving this potential” (Sterling et al., 2019, p. 5)	• As an ethical concept emphasizes the principle of *distributive justice* and is linked to human rights (Obasogie et al., 2017)
		• Pursuing health equity means, “removing obstacles for groups of people- such as the poor, disadvantaged, racial/ethnic groups, women, or persons who are not heterosexual- who historically have faced more obstacles to realizing their rights to health and other human rights.” (Braveman, 2006, p. 181)

### Health Disparities, Health Inequities, and Health Equity

#### Health Disparities

In a web page from the Centers for Disease Control and Prevention, HDs have been defined as “preventable differences in the burden of disease, injury, violence, or opportunities to achieve optimal health experienced by socially disadvantaged racial, ethnic, or population groups and communities” (Centers for Disease Control & Prevention (CDC), 2021). These differences reveal significant gaps when comparing a marginalized (minoritized) group with an advantaged group in terms of differences in rates of death or disease (Braveman et al., 2017). Around the year 2000, a paradigmatic shift occurred away from using the term *health disparities* (HDs) towards using the term *health inequities* (HI). The latter term considers how these differences are contextualized and interpreted under a social justice lens (Kneipp et al., 2018).

#### Health Inequities

HIs are similar but not synonymous with HDs. HIs are differences in death and disease that have been imposed systemically by “unequal access to social or economic resources” (Kneipp et al., 2018, p. 233). This approach asserts that HIs are created by unjust structural conditions that unfairly benefit a privileged social group while imposing multiple oppressive conditions and barriers on one or more minoritized and thus marginalized groups (Brownson et al., 2021; Shelton & Adsul, 2021; Trinh-Shevrin et al., 2015). The core question here is, “What is fair and just, and what is unjust, regarding access to essential opportunities and resources that ensure health and wellbeing?” Ultimately, HIs are the consequences of multiple upstream social determinants, such as poverty, social, economic, and educational barriers, that produce high rates of mortality, morbidity, and other poor health and mental health outcomes persisting across generations among many racially and ethnically marginalized groups (Groos et al., 2018).

#### Health Equity

Healthy People 2020 defines HE as “the attainment of the highest level of health for all people” (U. S. Department of Health & Human Services, 2019). This definition is aspirational in nature, with an abiding challenge on how best to assess incremental progress towards this lofty goal. Attaining this goal will require the removal of numerous obstacles to health and wellbeing, poverty, and discrimination; the inadequate access to basic needs; and the perpetuating forces of racism and prejudice (Braveman et al., 2017, p. 12; Koh et al., 2011; Obasogie et al., 2017). This analysis includes a social justice context that describes HE as, “the ethical and human rights principle that motivates us to eliminate HDs” (Braveman et al., 2017, p. 2).

## Equity-Related Theories and Models

In this section, we review select theories and models of systems-oriented approaches that describe and explain how social determinants of health, racism, and oppression interact to produce HIs. While current theories and models may differ in focus, those that depict directional pathways can aid in conducting deep-structure analyses (Resnicow et al., 2000) of HIs and how they can be modified by strategic interventions that are designed to achieve specific HE outcomes and goals.

### Ecological Systems Models  

Ecological theory and systems models describe the effects of the environments and social determinants on human development and health. Bronfenbrenner’s classic ecological model is credited as the first to examine human development from an ecological systems perspective. That model depicts multiple interactive factors that occur within complex ecological systems (Bronfenbrenner, 1979). However, many concentric circle ecological models (Bronfenbrenner, 1979; Sallis & Owen, 2015) do not identify specific pathways that likely produce targeted outcomes. Other more, recent ecological models have added new domains, such as the chrono and exo domains (Fish & Syed, 2018). Other more dynamic models and frameworks allude to processes that effect change, although still lacking clear pathways that show the influence of one factor on another. Trinh-Shevrin and colleagues present a population health equity framework (Trinh-Shevrin et al., 2015) that emphasizes a social justice perspective for changing macro- and meso-level structural determinants through social policies, also adding a life course perspective. Koh and collaborators elaborate on how social determinants of health can produce HDs (Koh et al., 2010), while Krieger describes how discrimination constitutes a major unjust social determinant that leads to adverse health consequences (Krieger, 2012). In summary, prior ecological systems models suggest the occurrence of complex and reciprocal influences on HIs and HE yet often lack specific pathways that depict meaningful effects among their factors. Accordingly, the need exists for a more informative ecosystemic framework that displays specific pathways that constitute mechanisms of effect that occur within a dynamic ecosystemic framework.

### Minority Stress Theory

Minority Stress Theory (MST) asserts that cumulative exposures to both distal and proximal stressors eventually produce impaired physical and mental health outcomes (Meyer, 2003). Discrimination against marginalized persons often results in significant stigma and distress. In accord with intersectionality, MST describes how race/ethnicity, low socioeconomic status, and chronic exposures to stressful community environments create a cluster of toxic social determinants that produce adverse health outcomes (Myers, 2009). MST has informed research on sexual minority populations and also applied in cross-cultural research. Although MST is a Westernized theory, its constructs, including gender-related stigma, lack of family support, and psychological distress, have been applied and validated in other cultures, such as in China among men who have sex with men (Sun et al., 2020).

### Critical Race Theory

Critical Race Theory (CRT) focuses on the construct of race in examining inequities that are embedded within social, political, and economic institutions, systems, policies, and practices. It asserts that contemporary disparities have deep “historical and sociopolitical roots” (Ford & Airhihenbuwa, 2010, p. S32). CRT proposes a framework for intervening on systemic racism and the social and historical contexts under which racism and discrimination evolved and are maintained (Volpe et al., 2019). Thus, attaining HE requires the elimination of structural racism by changing systems and structures to provide equal access to opportunities among communities of color to enhance health and wellbeing. A limitation of CRT is that it focuses primarily on the role of race/racism as a social determinant of HDs and HIs. Focusing on the single construct of race as the major social determinant misses the importance of adopting an intersectionality perspective.

### Intersectionality as a Theory

Intersectionality (Cresnshaw, 1989) is both a construct and a theoretical framework. Intersectionality as a theory describes the presence of multiple interlocking systems of privilege and oppression (Collins, 2015) that originate mostly at the macro level, yet impose detrimental effects at lower ecological levels among persons with various social identities. These identities include race, ethnicity, gender, age, sexual orientation, socioeconomic status, religious identification, and physical and cognitive ability. This intersection of these identity factors cumulatively can produce marginalization and higher risks for adverse health outcomes.

As a theory, intersectionality has evolved from its original conceptualization that focused on the oppression of Black/African American women. Crenshaw argued that the marginalization of Black women originates from the intersectionality of racism and sexism and that this type of intersectionality differs from the intersectionality experiences of White women and Black men. This approach emphasizes the need to understand how HIs differ at the intersection of various identities that are marginalized by a privileged majority.

## Application of Theories Related to Prevention Science

The aforementioned theories and models echo common themes from an ecosystemic perspective. They assert that unjust macro-level structural inequities operate synergistically to produce adverse health outcomes among people having various marginalized identities (Cooper & Christens, 2019). As noted previously, from a prevention science perspective, advancing HE will require designing interventions that change unfair and unjust structural determinants (Cox, 2020) by addressing structural and systemic racism, sexism, classism, ageism, ableism, gender, religion, and other dominant identity-related prejudices. Integrating these theories and their supportive evidence can inform in-depth multi-dimensional analyses of complex real-world issues. These analyses are important for incorporating scientific evidence conceptualized under an intersectionality lens into preventive intervention planning, design, and development to advance HE.

## Empirical Intervention Research with Implications for Health Equity

The prevention science field has established a strong evidence base consisting of prevention theory, methods, and interventions to improve health and wellbeing. It is now imperative to apply this foundation toward the design and development of equity-focused preventive interventions. In this section, we review select examples of EBPIs organized by ecological levels that illustrate applications of prevention science toward advancing HE. However, before this review, we seek to discuss the importance of community-engaged participatory research as a basis for viable HE research approaches.

## Engagement Approaches to Promote Health Equity

Engaging and empowering community stakeholders is critical for the successful development, implementation, dissemination, and sustainability of interventions that focus on HE change. Community-based participatory research (CBPR) and the authentic engagement of community partners (Okamoto, Kulis et al., 2014) is essential for HE advancement. This process includes engaging representative population partners in the co-creation and evaluation of EBPIs (Wallerstein & Duran, 2006). CBPR activities can empower community partners to prioritize their views and preferences, while taking into account community histories of racism and oppression (Parker et al., 2020). The process of co-creation fosters community ownership that, in turn, increases an EBPI’s acceptability, adoption, and sustainability within participating communities (Alvidrez et al., 2019).

### Macro-Level Interventions

Macro-level interventions can be qualitatively different in form and approach from evidence-based interventions (EBPIs) often conducted at the micro-level, whereas macro-level interventions often target policy-related or structural conditions needing change. Two particularly relevant policy realms for prevention science efforts are (a) policies designed to change social structures such as an intervention to raise the minimum wage to reverse poverty conditions, and (b) policies focusing on population-level behavior changes, such as taxes on tobacco, alcohol, and unhealthy foods (Van Ryzing et al., 2018).

In an analysis of state wages in the USA during the year 2010, Black mothers living in states with a higher minimum wage exhibited significantly lower infant mortality rates when compared with Black mothers living in states having a low minimum wage, although this finding was not observed among White mothers (Rosenquist et al., 2020). In this case, race (Black, White) appears as a moderator of this differential outcome. In another analysis focusing on the effects of minimum wage on birth outcomes among adolescent girls, a higher minimum wage was associated with lower pre-term birth rates among Latina and White adolescents, but not for Black adolescents (Bullinger, 2017). These two studies suggest the potential benefits of a structural change in minimum wages whereby a fair minimum wage could advance HE among certain marginalized groups. Nonetheless, given the fact that all marginalized groups did not benefit the same way, it suggests the need to conduct deeper analyses to understand these effects.

At the macro level, *structural interventions* are another means of changing specific social determinants that constitute upstream drivers of health inequities, such as the dissemination of condom bowls, educational flyers, and media programs to teach condom use. A meta-analysis of condom distribution interventions has shown that these approaches are effective in health promotion with women sex workers (Charania et al., 2011). Community-level behavior change interventions have the potential for large-scale implementation to attenuate unfair differences in opportunities and resources among marginalized groups, thus constituting a form of restorative justice (Cooper & Christens, 2019).

### Meso-Level: Community-Based Approaches

At the meso level, structural community-based interventions can aim to eliminate local barriers for accessing essential resources, such as health care or school attendance, through interventions implemented within community settings (e.g., community health centers, schools, grocery stores, and home-based outreach). Community engagement strategies are critical to producing interventions that promote health equity at the community level based on shifting the power gradient from the researchers to community representatives.

### Micro-Level: Familial and Individualized Approaches

Established individual and family-oriented preventive interventions exist that are efficacious in addressing common health problems such as obesity, diabetes, and family dysfunction. Currently there exits limited evidence on the effects of prevention interventions that are explicitly designed to reduce HDs and HIs. Few studies have been specifically evaluated or have sufficient sample sizes and power to examine various HE outcomes.

In the following section, we review common types of preventive interventions that operate across macro, meso, and micro levels. These include school-based interventions, early childhood home-visiting, obesity and diabetes prevention, suicide and substance misuse preventive interventions that are delivered with communities, community sub-groups, families, and individuals. We review both the strengths of these approaches to promote HE and their deficits that need to be modified with more effective prevention science approaches designed to address HIs and promote HE.

#### School-Based Interventions

##### Universal Prevention Within Multicultural Environments

Over the past 30 years, a solid evidence base has been established on school-based substance use preventive interventions. For example, *Life Skills Training * is an established universal school-based substance use prevention curriculum having demonstrated efficacy with youth of color (Botvin et al., 2001). Similarly, *Positive Action * is a universal school climate and youth-focused prevention intervention having demonstrated efficacy with youth in Hawaii—a state populated predominantly with Asian Americans and Pacific Islanders (Beets et al., 2009).

##### Culturally Focused Resistance Skills Training

There exist several school-based preventive interventions developed with the inclusion of the cultural and community norms from the youth they intend to serve. This approach combines scientific efficacy with community acceptability, increasing the probability for uptake and effective real-world implementation. The most established example is the original *keepin’ it REAL* drug use refusal skills training adapted for Mexican/Mexican American youth in the Southwest (Hecht et al., 2003). *Keepin’ it REAL* refusal skills were also adapted in the *Living in 2 Worlds* studies program for American Indian/Native American youth (Kulis et al., 2013). It has also been adapted in the Ho ‘ouna Pono studies for Native Hawaiian youth (Okamoto et al., 2016, 2019).

##### EBPI Efficacy Limits Suggest Need for Structural Changes

School-based prevention withyouth of color has focused on youth- and school-level changes. Nonetheless, the need has emerged for addressing meso-level factors (e.g., contextual community factors) to produce complementary structural changes in school and local community environments. For example, the need emerged to consider the density of community alcohol outlets that contribute to underage alcohol use. A higher density of alcohol outlets has been observed within low-socioeconomic neighborhoods that are populated by people of color (Lee et al., 2020). From an ecological multi-level perspective, it became evident that focusing solely on youths’ individual skills was insufficient for complete substance use prevention. Needed also was a complementary meso-level intervention component that would change community-level conditions by introducing safeguards that restrict youth access to alcohol and tobacco within several local community outlets.

##### EBPI Curriculum as an Adaptations “Anchor” for Similar Cultural Groups

Culturally focused, school-based prevention is still in its initial stages of development. Time and resources do not permit developing a school-based prevention program for each of several specific racial/ethnic groups across the USA. In response, Okamoto, Helms et al. (2014) proposed a regional cultural approach whereby school-based prevention curricula for specific cultural groups could serve as an anchor for EBPI adaptations to accommodate other cultural groups that share similar cultural and regional characteristics. For example, the Hawaiian Ho ‘ouna Pono substance use refusal skills training curriculum has been adapted for Filipino, Marshallese, and Chamorro youth in Guam.

#### Home Visiting Interventions for Maternal and Child Wellness

A well-established evidence base has also emerged for home visitation programs that support new and expectant mothers and their young children who live in underserved and high-risk communities. A review of research in the USA from 2005 to 2015 examined 39 studies on home-visiting and found this approach to be effective in helping individuals from HD groups to “avoid injury, maintain health, and prevent and manage disease” (Abbott & Elliott, 2017). However, this article fell short of reporting details on whether the home-visiting interventions produced better, worse, or the same effects among racial/ethnic subgroups when examined against the majority or health-equitable comparison groups.

Some trials have identified how racial/ethnic subgroups within various study populations are differentially affected by a home-visiting intervention. For example, a study of the *Mind the Baby* home-visiting intervention conducted with a diverse population of medically underserved expectant mothers showed an overall positive effect at age two on their children’s healthy weight status. The best outcomes were seen within a subgroup of participating Hispanic children whose obesity risks were higher than those of the other racial groups in this study (Ordway et al., 2018). Similarly, a *Healthy Families America* (HFA) home-visitation study was conducted to reduce child maltreatment among low-SES women by reducing harsh parenting. When comparing three subgroups, Spanish-speaking Latina mothers, English-speaking Latina mothers, and non-Latina Caucasian mothers, the Spanish-speaking Latina mothers, despite their lower SES, exhibited fewer harsh parenting behaviors as compared with the other subgroups (Martin et al., 2012). However, to determine changes in HE, neither the *Mind the Baby* nor the HFA studies compared the gains of the subgroups to majority reference populations.

Toward this end, a California study examined birth outcomes among mothers (*N* = 1102) in a region with high Medicaid participation who received the *MOMS* home-visitation program. In comparison with general population samples from the local region and state of California who did not experience *MOMS*, Hispanic mothers receiving this program exhibited greater program benefits than non-Hispanic white mothers who received *MOMS* (Guo et al., 2016). Meanwhile, two home-visiting trials conducted specifically with high-risk African American mothers exhibited negative results (Thomson et al., 2018). Reinforcing the importance of CBPR to promote HE, neither study provided evidence that the study populations’ needs, preferences, cultural values, and beliefs had been incorporated into the interventions and their evaluation. Furthermore, both studies focused on reducing risk factors, rather than increasing protective factors. Unfortunately, risk-only approaches that do not promote protective factors may fail to motivate participants from culturally distinct and historically disenfranchised populations (Borowsky et al., 1998).

From a HE perspective, this review of the home-visiting literature underscores the importance of engaging communities to participate in the co-design of home-based interventions. Doing so addresses the contextual needs and priorities of the diverse racial/ethnic populations being served. Taking this one step further, *Family Spirit*, whose content was designed and positively evaluated for young Native American families is now testing a precision approach that customizes content to the needs of diverse individual families who live in Native American communities (Haroz et al., 2020). *Family Spirit* researchers have also used measures for Native American children’s outcomes that can be compared to normative reference populations to examine incremental increases in HE (Barlow, 2022).

#### Prevention of Obesity and Type 2 Diabetes

Within the USA, obesity and type 2 diabetes (T2D) constitute major public health problems (Kumanyika, 2019). Compared with the non-Hispanic White Americans, rates of obesity and T2D are higher among Black/African American, Hispanic/Latinx, and Native American populations (Centers for Disease Control & Prevention (CDC), 2020). The *Diabetes Prevention Program* (DPP) and its adaptations are individually-focused lifestyle interventions developed for diabetes prevention with African American, Latinx, and Alaska Native/American Indian populations or subgroups (Diabetes Prevention Program Research Group, 2002). The original DPP consisted of a comprehensive study of 3234 adults, with this sample consisting of Blacks/African Americans (19.9%), Hispanics/Latinxs (15.7%), Native Americans/American Indians (5.3%), and Asians (4.4%) (Diabetes Prevention Program Research Group, 2002). This preventive intervention has been effective as assessed by several randomized controlled trials (RCTs) conducted with several racial/ethnic groups (Knowler et al., 2002).

Culturally adapted versions of the DPP developed for urban African American families with children have shown improvements on body mass index (BMI) and health-related behaviors (Burnet et al., 2011). For middle-aged Latinas, improvements have been observed on diet, weight loss, BMI, glucose/insulin levels, stress, and depressive symptoms (McCurley et al., 2017; Sorkin et al., 2014). For Native American youth and adults, improvements have been observed on healthy changes in BMI, quality of life, hypertension, HbA1c, physical activity, and nutrition (Brown et al., 2010; Kenney et al., 2016; Sauder et al., 2018). Finally, for Filipino American populations, improvements have been shown for weight loss, diet, physical activity, and cholesterol (Bender et al., 2016).

Common factors in the process of adaptations of the DPP included gathering qualitative data from key community partners and community residents, incorporating culturally-specific preferences and practices into the conceptual model and intervention components, and having members from the community serving as *promotoras* (lay health workers), who delivered the intervention. One limitation is that individual EBIs such as the DPP have not targeted changing local community meso-level structural factors, such as eliminating food deserts, that operate as structural conditions that can elevate risks for diabetes and obesity (Walker et al., 2014).

#### Parenting Interventions to Prevent Youth Behavioral Health Problems

Promoting positive parenting and family functioning are approaches for effecting positive outcomes among youth (Coatsworth et al., 2002). Two interventions have established an evidence base as a culturally-tailored interventions applied with specific racial/ethnic families and their children. First, the *Strong African American Families* is a family-based preventive intervention designed for rural African American families who have a pre-adolescent child. It focuses on parenting, family communication, and improving both child and parent competencies. This intervention has been effective in reducing substance use and misuse, depressive symptoms, and youth conduct problems and has demonstrated sustained long-term effects (Brody et al., 2010, 2012).

Second, *Familias Unidas* is a family-based intervention for Latinx families that focuses on parenting within the context of the immigrant experience while empowering parents and promoting skill-building for both parents and youth. When compared to community practice control groups, *Familas Unidas* has been effective for a range of problems that include reducing and preventing substance use, externalizing and internalizing behaviors, and unsafe sexual behaviors (Pantin et al., 2009; Perrino et al., 2014; Prado et al., 2012).

Both of these EBPIs were designed for a specific racial/ethnic group with intervention implementation presented within the context of a local ecological system—the family system in *Familias Unidas* and the local community in the *Strong African American Families* program. These interventions help individual youth within the contexts of their family, schools, and the local community. Although focusing on individual children and parents, this broader systems approach is consistent wth HE goals. These interventions aim to strengthen familial protective factors to prevent behavioral and substance use problems. One limitation is that systemic stressors and structural conditions that place ethnocultural families at risk are not explicitly addressed in family-based EBPIs such as these (Brody et al., 2012; Hawkins et al., 2015; Khare et al., 2009; Office of the Surgeon General, 2016; Prado et al., 2012).

#### The Celebrating Life Project

The *Celebrating Life* intervention is a multi-component preventive intervention that includes a tiered package of universal, selective, and indicated intervention components (Kellam & Langevin, 2003). This intervention was co-created by the White Mountain Apache Tribe and Johns Hopkins research collaborators (Cwik et al., 2016). The core of this approach consists of a tribally mandated suicide surveillance and case management system that employs local Apache paraprofessionals to follow up on every reported event (suicide attempt, ideation, binge substance use, non-suicidal self-harm) to assess imminent risks and connect at-risk individuals to local care. In addition, this system is supported by local public education campaigns, workshops for tribal members to identify and act on signs and symptoms of suicide among youth and other community members, a curriculum for middle school students to promote protective cultural factors, and the inclusion of elders as wise purveyors of cultural knowledge and support.

The White Mountain Apache’s tribally mandated suicide surveillance system was used to compare suicide attempts and deaths over two six-year time periods that occurred before and after the comprehensive *Celebrating Life* case management and community education campaigns were launched. Findings of *Celebrating Life *revealed a 38% decrease in suicide deaths and a 53% reduction in suicide attempts between the two time periods, as these were compared to national data. These data showed slightly increasing suicide rates among all US and all American Indian/Alaska Native populations. (Cwik et al., 2016).

These results provide initial evidence that when compared with the general population, the *Celebrating Life* intervention reduced the suicide disparity rates among the White Mountain Apache population. These findings suggest a reductions in HI that also advance HE. The quasi-experimental design utilized in the *Celebrating Life* study could be regarded as reducing the study’s *internal validity* when compared with utilizing a randomized controlled research design. By contrast, the *Celebrating Life* study was designed for high *external validity* based on its real-world community focus that invited and benefited from the active participation of the White Mountain Apache community who participated in the study’s co-design and implementation. This higher external validity was further enhanced by engaging tribal elders to participate as important community advisors.

#### The Qungasvik Project

A second exemplary multi-level intervention is *Qungasvik* (toolbox), a strength- and community-based cultural campaign developed by using a CBPR approach. For Yup’ik adolescents ages 12 to 18, this study examined protective factors for preventing suicide and alcohol use at differing ecosystemic levels: individual, family, peers, and community (Philip et al., 2016). In a related intervention study (Allen et al., 2018), the effects of a more intense version of the *Qungasvik* intervention in Community 1 were compared with a less intense version in Community 2. In a four-wave community analysis, investigators report greater growth in protection against suicide in Community 1 versus Community 2. This intervention provides protection against suicide as indicated by a significant growth curve over time in Community 1, but not in Community 2, and as assessed by a specific mediator variable. This mediator variable, *Reasons for Living*, measured culture-specific beliefs and experiences that make life enjoyable and worthwhile within a rural Yup’ik context. Whereas this multi-level intervention study has some limitations, it illustrates how a sufficiently rigorous quasi-experimental design and an intervention specifically tailored for Indigenous communities can exert significant effects in protection against suicide.

#### Key Features of Multi-Level Interventions

Presently, interest is growing in multilevel research designs that consist of preventive interventions that emphasize greater external validity (Leviton, 2017). These designs can yield significant results under a complex real-world implementation. As noted, the *Celebrating Life * and *Qungasvik* community-based interventions exhibited greater external validity in design and intervention components, which targeted beneficial changes in community-level factors. In *Celebrating Life*, these components included local community mental health case managers who followed-up with at-risk individuals, wherever they could be found, to connect them with available and preferred care. In the *Qungasvik* intervention, the community-level approach consisted of a comparison of differing intensity levels of intervention delivery as presented within two communities. Both *Celebrating Life *and *Qungasvik* were conducted with American Indian and Alaska Native communities. These exemplar interventions may pave the way for the future development and implementation of sufficiently rigorous and efficacious multi-level preventive interventions that are high in external validity and in scale-up readiness.

Progress towards advancing HE often requires multi-level approaches. This implicates a paradigm shift (Blue Bird Jernigan et al. 2020; Cwik et al., 2016) away from conventional RCT interventions that maximize *intervention efficacy* (greater internal validity), instead focusing on *intervention effectiveness* in its delivery within real-world community contexts (greater external validity) (Skivington et al., 2021). This multi-level approach also recognizes that strategies to advance HE are qualitatively different when implemented within or across each of the three ecological domains (macro, meso, and micro) (Kok et al., 2015).

## An Ecosystemic Framework for Promoting Health Equity

### Prevention Science Through a Health Equity Perspective

**Fig. 1:**
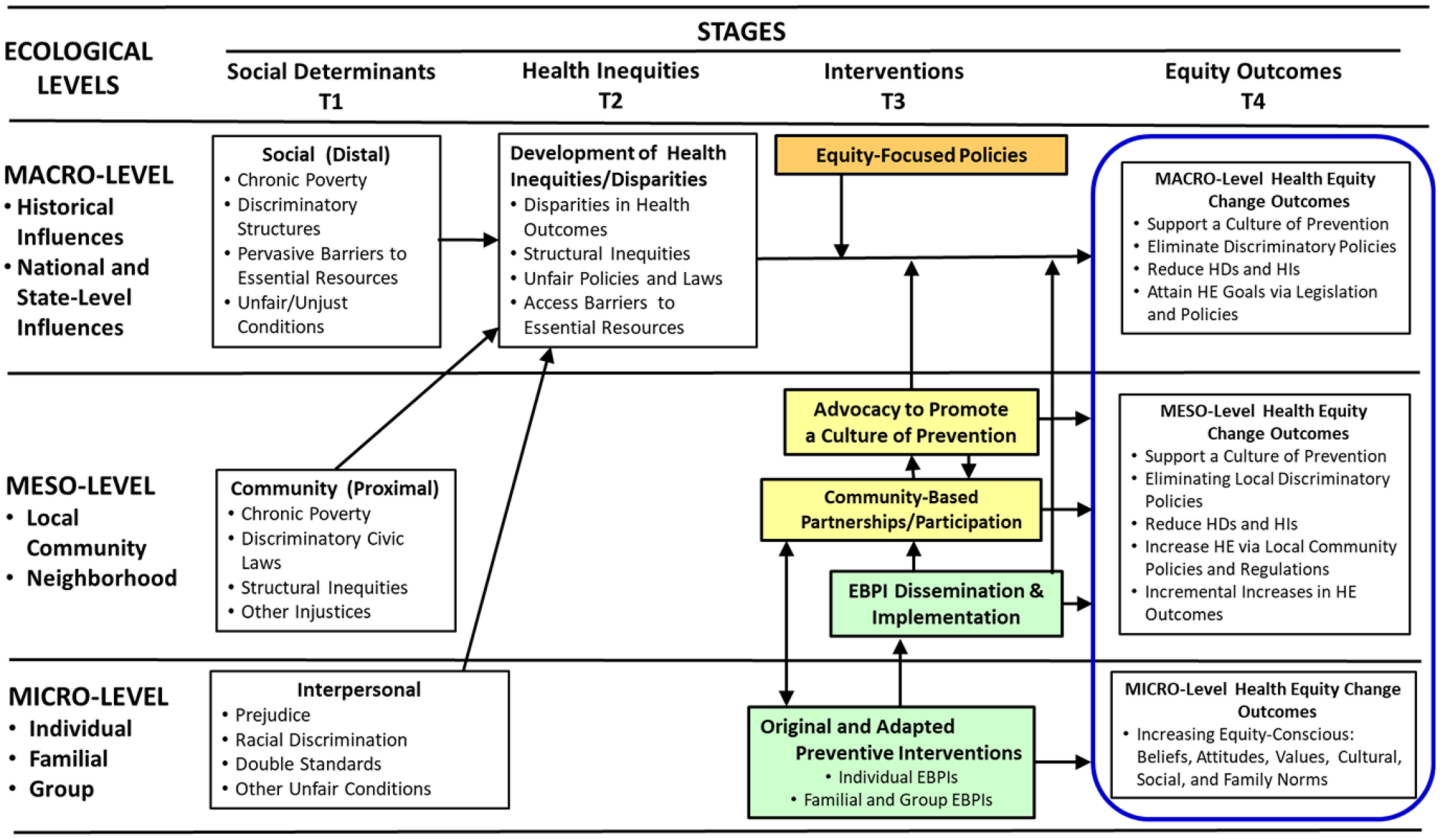
Ecosystemic framework of pathways to advance health equity

In Fig. 1, we introduce the *Ecosystemic Framework* for conceptualizing major pathways in which unjust social determinants produce HDs and HIs. In our framework, we extend Bronfenbrenner’s classic ecological framework (Bronfenbrenner, 1979) and the work of other ecological models and frameworks (Boyas et al., 2017; Dunkel Schetter et al., 2013; Pantin et al., 2004; Trinh-Shevrin et al., 2015). Our *Ecosystemic Framework* advances beyond prior ecological frameworks by adding specific pathways of stagewise effects (Castro et al., 2009). These effects aid in describing known or expected mechanisms that operate as drivers of equity outcomes. This analysis also prompts the application of strong theories and best empirical research for explaining these mechanisms to the fullest extent (Gottfredson et al., 2015). This *Ecosystemic Framework *can guide the analysis and interpretation of several specific pathways as components of a larger sociocultural process. This analysis can thus inform the planning and application of multi-level preventive interventions. This framework allows a more detailed analysis of specific pathways (mechanisms of effect) examined within the context of three ecological domains (macro, meso, micro).

### Ecosystemic Framework and Applications for Prevention Science Research

In our *Ecosystemic Framework* (Fig. 1), macro-level mechanisms or processes consist of national and statewide policies, legislation, and broad structural interventions. Meso-level processes focus on local communities and their municipal policies, legislative initiatives, and structural interventions. The micro-level focuses on processes that occur among individuals, families, and groups. In a “walk-through” of this stagewise temporal process, our *Ecosystemic Framework* shows that at time (T_1_) unjust social determinants such as discriminatory laws, social discrimination, and barriers to essential resources can operate as “root causes” that constitute upstream drivers. These structural determinants at the macro and meso-ecological levels produce HDs and HIs (at T_2_). These HIs can be attenuated at (T_3_) with efficacious interventions implemented at one or more of the three ecosystem levels. These interventions when applied in synergy at (T_3_) can yield one or more specific equity outcomes at (T_4_).

In particular, multi-level interventions are those designed for coordinated implementation across two or three of these ecological levels (domains) (see “Key Features of Multi-Level Interventions”). Their synergistic effects could be designed to produce short-term incremental changes that constitute short-term HE outcomes (Barlow & Okamoto, 2022). In principle, multi-level interventions can be designed to contribute to a *culture of prevention* (Fishbein, 2021) to produce HE outcomes (at T_4_). For a given HI, a coordinated intervention process could consist of (a) macro-level equity-focused policies, (b) meso-level community advocacy and action, and (c) the implementation of a problem-specific intervention at the micro-level. This *Ecosystemic Framework* illustrates three distinct pathways that can be designed for such synergistic efforts to reduce HIs and advance HE. It can be noted that across each of these ecological domains, relevant equity outcomes may be similar in focus, yet different in their form based on the ecological domain (macro, meso, micro) in which this stagewise process is implemented (see equity outcomes in Fig. 1).

### Ecosystemic Framework Macro-Level Preventive Intervention Pathway

At the macro level, prevention science can engage policymakers through advocacy and scientific consultation (the orange pathway) to support *Equity-Focused Policies* that allocate funding and sociopolitical support introduced at the macro-level to establish and sustain a culture of prevention and eliminate discriminatory policies to reduce HI and advance HE. A culture of prevention can be created by establishing social and cultural norms that are acceptable and practiced by residents of a local community (Fishbein, 2021; Gottfredson et al., 2015) to create an every-day culture of prevention in the lives of community residents. This can also involve collaborations between prevention scientists, policy makers, and the public (Fishbein, 2021). A related macro-level intervention is advocacy to promote legislation that supports funding for sustaining preventive interventions and for training new cohorts of prevention scientists who can design, develop, disseminate, and evaluate these preventive interventions (see Recommendation #6).

### Meso-Macro-Level Preventive Intervention Pathway

An intervention can originate at the meso domain among community partners, community organizations, and prevention scientists. Under this partnership using community-based participatory research (CBPR) principles (Israel et al., 2018), collaborators can co-design an equity-focused intervention for implementation at local community levels (the yellow pathway). This can also include collaborations with legal scholars to define and address complex issues involving social justice in advancing HE. Minkler and colleagues assert that “there can be no health equity without racial equity and social justice” (Minkler et al., 2019). Similarly, based on the social issues addressed, the field of prevention science can establish strategic equity-focused collaborations with scientists and professionals from public health, implementation science, and systems science, as well as with coalitions, such as the National Prevention Science Coalition. This leadership can extend to the macro domain by collaborations to create policy-based research to support the co-creation of community action projects to promote structural changes that advance HE within the meso and macro domains (Kegler et al., 2019).

### A Micro-Meso-Macro-Level Preventive Intervention Pathway

At the micro level, prevention science can extend what it does best—developing efficacious EBIs (green pathway) implemented at the micro domain and explicitly designed for *scale-up readiness* (Fagan et al., 2019) for their dissemination and implementation in the meso domain. Ideally, these EBPIs are designed with a built-in capacity to facilitate their adaptations at the meso level, for greater cultural relevance and fit with the goal of ensuring broad scale and seamless adoption, adaptation, dissemination, and implementation within each of many diverse communities (Castro & Yasui, 2017).

## Considerations and Applications of Strategic Directions for Advancing Health Equity

This section examines considerations and novel applications for conducting deep-structure analyses to support innovative research and intervention planning for advancing HE.

## Some Application Issues

### Issues in the Conceptualization and Measurement of HE

To make verifiable increments in HE, a great need currently exists for the development of reliable and valid measures of HE outcomes (Brownson et al., 2021). Efforts to operationalize and measure HE have met notable challenges (Penman-Aguilar et al., 2015, p. S 35). First, HE, HIs, and HDs are complex social justice constructs that originated from legal discourse on social justice (Kneipp et al., 2018). Second, HIs are not just defined as differences but also as direct consequences of unfair social conditions (Penman-Aguilar et al., 2015). Accordingly, these constructs have more complex measurement properties than do concrete physical measures such as height and weight. This is because the meaning of particular HIs and HE is influenced by the environmental and social justice contexts in which these constructs are examined (Kneipp et al., 2018; Penman-Aguilar et al., 2015). Thus, it is urgent to develop valid and reliable equity-relevant metrics of these complex social justice constructs (Brownson et al., 2021). One strategic approach is to identify variables that constitute reliable and valid indicators of HE. These measures must be able to assess incremental changes in HE to facilitate the tracking of ongoing progress towards attaining a major HE goal. For example, among marginalized individuals and families, one HE goal could be to close the health care gap between the health care needed and the availability of fair access to needed care (Tapager et al., 2022).

### Cultural Factors: Use in Models to Increase Cultural Relevance

The communities most affected by HDs and HIs are racial/ethnic minoritized communities. Despite this reality, a pervasive gap in the literature on HDs and HIs and their related frameworks and models involves an absence of cultural factors in many models, frameworks, and analyses. One remarkable example of this pervasive omission is that among 73 implementation strategies developed by the Expert Recommendations for Implementing Change (ERIC) (Powell et al., 2015) not a single strategy mentions culture or cultural factors. The analysis of culture has been described as fundamental for effective intervention implementation among indigenous peoples (Barlow & Okamoto, 2022; Manson, 2020). Of course, this also applies to other racial/ethnic groups including Hispanics/Latinxs and Blacks/African Americans.

Cultural factors are constructs that capture core aspects of an ethnocultural group’s cultural values, beliefs, and lifeways (Castro & Hernandez-Alarcon, 2002). Culturally, common factors are those applicable across more than one major ethnocultural group. These include acculturation, ethnic pride, traditionalism, and collectivism (versus individualism). For example, within Hispanic/Latinx communities, major cultural factors include acculturation stress, bicultural identity, *familismo* (familism), *personalismo* (the value afforded to personal relationships), *respeto* (respect for elders and persons of higher social position), and *simpatia* (the importance of courtesy in interpersonal relationships) (Castro & Hernandez-Alarcon, 2002; Castro & Kessler, 2021; Ramirez Garcia, 2019).

The field of prevention science needs to advance the understanding of how cultural factors can be operationalized into variables, measured and assessed using quantitative or mixed methods approaches (Creswell & Creswell, 2018). This also recognizes the importance of qualitative approaches that can capture the thick rich aspects of culture that cannot be fully captured with quantitative methods. This said, quantitative-measured cultural factors can be incorporated into multivariate models to assess cultural effects on targeted outcome variables. Cultural factors may operate as predictors (antecedent factors) or as moderators or mediators of effect. Regarding the role of cultural factors in HIV risk behaviors, Lightfoot and Milburn (2009) asked, “How can culture be incorporated into HIV interventions to reduce HIV-related risk behaviors among African-American youth?” Among these youths and their families, investigators examined the Black/African American cultural factors of Afrocentric values, racial socialization, and racial/ethnic pride.

### Intersectionality: Considerations and Analytic Approaches

As noted previously, intersectionality is a framework, theory, and approach that examines the interactive effects of several identity variables. Three of the most often examined identity variables in intersectionality analyses have been gender, LGBT identities, and racial/ethnic identities (Parent et al., 2013). From an intersectionality perspective, the synergistic effect of several marginalized identity factors such as having an ethnocultural identity, being a woman and single parent, and living in a community affected by high rates of crime and violence can produce high levels of acute and chronic stress and risks of anxiety, depression, and diagnosable trauma. From a rights-based approach, identity factors can be examined within the context of human rights to inform the development of health policies and intervention programs designed to promote HE. These factors can be defined under a social justice lens towards improving quality of life and increasing life chances for upward social economic mobility (Taket & McKay, 2022).

Empirical intersectionality analyses can examine complex constructs such as attaining “Gender Equity” by developing fair policies that will reduce HIs (Mena & Bolte, 2019). Intersectionality-based quantitative data often examine gender-by-race interaction effects in multiple regression model analyses. By contrast, under a qualitative approach, intersectionality analyses often describe the oppression, marginalization, and vulnerability experienced from exposures to unfair real-world conditions. When framed under a social justice lens, unfair power structures are examined as social determinants of HDs and HIs, such that social actions are necessary to rectify these injustices (Abrams et al., 2020). From a methodological perspective, qualitative and mixed methods procedures are best utilized for conducting deep-structure analyses of these complex real-world conditions.

### Building on Concept Mapping, Intervention Mapping, and Logic Models

Concept mapping and intervention mapping approaches are useful tools for identifying core treatment and intervention components. Using a CBPR approach, Green and colleagues interviewed community agency collaborators to conduct concept mapping. Using brainstorming sessions, these collaborators developed a “conceptual framework for guiding… theory-informed intervention development, planning, and organization” (Green et al., 2012, p. 364). Their concept mapping procedure consisted of six stages: preparation, generation, structuring, representation, interpretation, and utilization (Green et al., 2012).

Similarly, Byrd and colleagues applied a CBPR approach with lay health workers and community advisory board members by conducting focus groups to elicit participants’ views. The goal was to identify intervention determinants to inform the development of a culturally appropriate and theoretically sound intervention for cervical cancer prevention for women of Mexican heritage. Their intervention mapping approach consisted of four steps: (a) conducting a needs assessment, (b) creating matrices of program objectives, (c) selecting intervention methods and strategies, and (d) designing the program (Byrd et al., 2012).

The *Implementation Research Logic Model *(IRLM) has been developed for conducting dissemination and implementation analyses and planning (Smith, 2022; Smith et al., 2020). The Disparities-Equity Task Force developed the Ecosystemic Framework independently of the IRLM, although both frameworks can serve as resources for analyses that inform the development of health equity statements, as described below. IRLM analyses consist of four parts: (a) identifying implementation determinants that constitute barriers or facilitators; (b) identifying and describing implementation strategies, (c) describing mechanisms of action, and (d) identifying intended outcomes. For greater depth of analysis in applying the IRLM with racial/ethnic and other minoritized communities, cultural factors can be included into these model analyses.

## The rationale for Developing a Health Equity Statement

In this section, we introduce an analytic approach for conducting in-depth analyses to advance research for increasing health equity (HE). We refer to this analysis as a *Health Equity Statement * (HES). Its purpose is to serve as a *living document* to aid in expanding our understanding of ecological processes that have created health inequities (HIs) and of counter processes that can attenuate or eliminate HIs and increase HE.

Guided by our *Ecosystemic Framework*, a Health Equity Statement consists of a text narrative analysis that applies theory and empirical evidence in describing the role of social determinants, mechanisms, and interventions as influences that can yield specific HE outcomes. A well-developed Health Equity Statement can “advance our understanding of social determinants of health and pathways through which they operate on specific health outcomes” (Penman-Aguilar et al., 2015, p. S40). This includes the analysis of social justice as an influence in affording marginalized or minoritized groups with fair conditions for accessing health care and other essential resources (Sterning et al., 2019).

Using the Ecosystemic Framework as a guide, a Health Equity Statement systematically examines each of several ecosystemic pathways (mechanisms of effect) for an integrative analysis of multiple pathways (processes) that describe temporal stagewise effects that can yield specific HE outcomes and a HE goal. In this integrative analysis, scientific theories and best research-based empirical evidence are to be incorporated into a Health Equity Statement. This can also aid in advancing theory in describing major pathways that can reliably produce targeted HE outcomes. In this endeavor, mechanisms having strong evidence based on established knowledge can be recognized as solid evidence. Other mechanisms supported by partial empirical evidence may be described as “likely” effects. Other less, certain mechanisms may be described as “possible” effects. And pathways of mechanisms having much uncertainty can still be described albeit identified as “gaps in our knowledge,” thus revealing opportunities for generating research that produces the new knowledge needed to inform this gap.

A health equity statement may be regarded as a living document, whereby iterative revisions would address gaps revealed in a prior analysis. Finally, a modified version of a health equity statement can be written to inform legislators, community residents, and others about strategic approach mechanisms that can be used to resolve a HE problem in a statement that is tailored for a particular audience.

## An Exemplar for Developing a HE Statement: Black Youth Suicide Risk

In constructing a health equity statement to address Black youth  suicide risk, social determinants of health at the micro, meso, and macro levels should be considered. At the micro level, exposure to higher levels of interpersonal discrimination for Black youth has been associated with their increased suicidal thoughts and attempts. This is particularly concerning, as recent research has found that these youth reported higher levels of discrimination than all other racial/ethnic groups (Argabright et al., 2022). At the meso level, it has been shown that neighborhoods with fewer college-educated residents were associated with greater suicide deaths among Black young  males (Votruba & Kling, 2009). Finally, at the macro level, a recent meta-analysis found that an increase in anti-Black racism  at the state level diminished the efficacy of mental health treatment for Black youth (Price et al., 2022). Racism and neighborhood economic deprivation are among the complex set of factors that can lead to increased suicide deaths and attempts among Black youth (Sheftall & Boyd, 2022).

Evidence-based prevention intervention strategies aimed at interpersonal and systemic racism, as well as improving youths’ economic conditions, must address multiple ecosystemic levels to promote health equity for Black youth. Addressing Black youths’ elevated suicide risk requires tailored, multi-level approaches that move beyond standard suicide prevention techniques (e.g., identification of suicide risk, youth emotion regulation skills, and/or communication education about warning signs). Prevention intervention strategies at the micro, meso, and macro levels are needed to address Black youths’ disproportionate suicidality rates and promote HE. These might include culturally grounded intervention strategies developed from the values and beliefs of Black youth within a specific regional and cultural context (Okamoto, Helms et al., 2014). These strategies can involve the community and policymakers to prioritize and address the problem, and implement tailored, culturally relevant, evidence-based strategies for these youth.

## Developing Health Equity Statements

As a living document, a health equity statement would “map out” evidence-based pathways examined within the context of our Ecosystemic Framework. This HES can build on prior instruments that include *concept mapping* (Green et al., 2012), *intervention mapping* (Byrd et al., 2012), and *logic models* such as the Implementation Research Logic Model (Smith et al., 2020). Its form as a text narrative will allow a Health Equity Statement to present a flowing description of complex effects, nuances, and contexts, as well as considerations and innovative approaches. A Health Equity Statement would aim to fill knowledge gaps about various pathways, as examined across stages of our ecological framework: (a) sociocultural *determinants*, (b) expected *mechanisms* of effect, (c) *interventions* designed to reduce inequities, and (d) specific *health equity outcomes*, i.e., short-term health equity outcomes and a long-term health equity goal.

The current exemplar HES describes inequities in suicide risks among Black youth, by identifying evidence in support of multilevel effects occurring at micro, meso, and macro-ecological levels. Such pathway analyses can be conducted for any of several public health problems that emerge as health disparities and inequities (Dankwa-Mullan et al., 2021). These analyses would also describe multilevel interventions (see Fig. 1), which can be informed by design and implementation intersectionalities among (a) *prevention science* which provides research on efficacious evidence-based preventive interventions (EBPIs) that can include *cultural adaptations* (Cabassa & Baumann, 2013; Castro & Yasui, 2017), and *cultural grounding* (Okamoto et al., 2019). These analyses can also be informed by (b) *implementation science* models and research on effective intervention transfer, adoption, and implementation (Aarons et al., 2011; Damschroder et al., 2009; Glasgow et al., 2019; Nilsen, 2015), and by (c) the application of *community-based participatory research* (CBPR) principles (Wallerstein et al., 2018) that can be implemented *in partnership* with community groups that have been adversely affected by health disparities and HIs (Orengo-Aguayo et al., 2020).

## Summary and an Invitation

In summary, a Health Equity Statement would consist of a rich integrative description and explanation of effects produced in hypothesized pathways that collectively drive a stagewise process that produces specific HE outcomes (see the equity outcomes column in Fig. 1). A health equity statement can provide a start-to-finish description of mechanisms of interest that constitute a total stagewise process of effects that produce specific HE outcomes or goals. One of these processes can be a stagewise analysis of EBPI design and delivery that is extended to examine mechanisms of EBPI dissemination and implementation. This particular analysis would thus combine two major stages: (a) the analysis of pathways of social determinants and intervention effects and (b) the analysis of pathways in effective EBPI dissemination and implementation. We recognize that the field of HE is still evolving (Penman-Aguilar et al., 2015). Accordingly, as living documents, Health Equity Statement may be modified on an ongoing basis, iteratively developing a progressively more complete analysis and explanation of mechanisms of effect as determinants of intended HE outcomes and goals.

Finally, the SPR task force’s Health Equity Statement broadly centers around addressing the micro-, meso-, and macro-level factors that allow adverse social determinants of health and health inequities to persist. These factors are best addressed using tailored EBPIs to be implemented with marginalized or underserved populations. This includes multi-level and/or structural interventions that can be successfully implemented and sustained within various communities (see Fig. 1). Woodward et al. (2019) have encouraged future research investigators to utilize their Health Equity Implementation Framework by inviting implementation scientists to extend their work. These investigators state, “we hope that scholars will apply and refine the framework we propose” (Woodward et al., 2019, p. 15). In accord with this spirit, based on analyses from our Disparities-Equity Task Force and guided by our Ecosystemic Framework, we invite prevention scientists and implementation scientists to utilize and improve our Ecosystemic Framework. One aim is to develop insightful health equity statements that explore, describe, and explain the pathways, mechanisms, and processes that will advance the strategic directions presented here, to expand our knowledge, theories, methods, and related contexts to yield a greater depth of understanding of how prevention science can advance HE.

## Recommendations

Based on the examination of the preventive intervention research and considerations of relevant theoretical models and the Ecosystemic Framework, the Disparities-Equity Task Force  put forth the following six recommendations to frame and promote future research to specifically redress HDs and HIs, and to advance HE.

### Recommendation #1: Adopt an Equity-Focused Approach for Developing New Prevention Science Interventions to Advance HE

In place of the conventional epidemiological disease framing of HDs and HIs, prevention science must adopt a proactive wellness-oriented equity focus for advancing HE by eliminating HDs and HIs. Our field must design new prevention interventions based on strategies that target equity-related constructs, such as social determinants of health and underlying systemic racism and other structural biases. Innovative design processes must consider environmental and other contextual factors to design and implement prevention interventions for sustained long-term impact. This approach can flourish with a close collaboration between prevention scientists who develop EBPIs and implementation scientists who specialize in the dissemination and implementation of interventions across diverse settings and with many populations.

### Recommendation #2: Refine the Ecosystemic Framework (Fig. 1)

The Ecosystemic Framework builds on the noted constructs and theories to identify social process pathways expected to produce targeted changes in equity outcomes (Fig. 1). This framework is put forth as a strategic road map for planning and organizing the development of multi-level interventions implemented across ecological levels with theorized pathways to promote HE change. Continuous modifications to this framework must be conducted on an ongoing basis to incorporate the latest and most robust empirical findings, for inclusion into innovative health equity statements.

### Recommendation #3: Develop and Implement Evidence-Based Multi-level Preventive Interventions

Large-scale multi-level structural interventions are key to producing comprehensive, coordinated, and synergistic equity outcomes across various eco-systemic levels. These interventions must move beyond individual impacts to include a strategic focus on modifiable structural and social determinants that drive and perpetuate inequities at differing ecosystemic levels. This includes advocacy and social action to garner the financial, political, and human resources needed to develop, implement, and sustain equity-focused structural changes for restorative justice in attaining equity outcomes at macro, meso, and/or micro levels.

### Recommendation #4: Establish Partnerships with Collaborators and Policy Makers

As noted, eliminating HDs and HIs to advance HE at a population level will require a coordinated implementation of EBPIs delivered at more than one ecosystemic level. Complementary health-related policies at the state, county, and local municipality levels will be needed for allocating and equitably distributing financial and political resources to sustain HE-focused EBPIs across time. This will require partnerships with diverse collaborators at all levels. Establishing these partnerships through CBPR and culturally grounded collaborations can become a gold standard for prevention science that advances HE.

### Recommendation #5: Expand Prevention Science Methodologies and Data Analytic Methods for HE Research

HE-focused prevention science must use and expand state-of-the-science data analytic methods to assess incremental and dynamic changes in HE while advancing toward a specific and greater HE goal. These improvements can include methods to evaluate intervention-related equity outcomes at all three levels: macro, meso, and micro. The field can also advance the development and use of common HD, HI, and HE outcome measures for assessing improvements in wellness and well-being. This also includes the development of methods and measures to examine, implement, and evaluate synergistic intervention effects of multi-level interventions across two or more of the ecosystemic intervention pathways.

### Recommendation #6: Develop and Support HE Training Programs in Prevention Science

Increased investment in training programs to support the development of new cohorts of prevention scientists and practitioners is essential to advance and sustain HE, especially in the inclusion of those who represent populations experiencing intergenerational HIs and HDs. Training programs should include existing prevention theories and methods that provide evidence-based knowledge and skills in scientific analysis, as well as content to increase the understanding and appreciation of cultural approaches for working effectively with diverse and marginalized population sectors. Due to inequities in research training, we must be intentional about incorporating the lived experiences, cultural insights, viewpoints, and world views that have been erased or marginalized by Western pedagogies. The goal is to produce a new generation of well-trained and prepared prevention science research investigators who can bring new energy, creativity, and cultural insights for developing innovative and efficacious multi-level interventions that will advance HE and reduce HDs and HIs. Prevention science also can contribute to the advocacy and development of training models for early-career prevention scholars from under-represented and marginalized communities.

## Abbreviations

HE: Health Equities
HD: Health disparities
EBPIs: Evidence-based preventive interventions
MST: Minority stress theory
CRT: Critical race theory
CBPR: Community-based participatory research
CDC: Centers for Disease Control and Prevention
DPP: Diabetes Prevention Program

## References

[CR1] Aarons GA, Hurlbert M, Horowitz LM (2011). Advancing a conceptual model of evidence-based practice in public health. Administration Policy and Mental Health and Mental Health Services Research.

[CR2] Abbott LS, Elliott LT (2017). Eliminating health disparities through action on the social determinants of health: A systematic review of home visiting in the United States, 2005–2015. Public Health Nursing.

[CR3] Abrams JA, Tabaac A, Jung S, Else-Quest NM (2020). Considerations for employing intersectionality in qualitative health research. Social Science & Medicine.

[CR4] Allen J, Rasmus SM, Ting Fok CC, Charles B, Henry D (2018). Multi-level cultural intervention for the prevention of suicide and alcohol use risk with Alaska Native youth: A nonrandomized comparison of treatment intensity. Prevention Science.

[CR5] Alvidrez J, Napoles AM, Bernal G, Lloyd J, Cartill V, Godette D, Cooper LA, Brave Heart MYH, Das R, Farhat T (2019). Building the evidence base to inform planned intervention adaptatoins by practitioners serving health disparity populations. American Journal of Public Health.

[CR6] Argabright ST, Vioski E, Moore TM, Ryna DT, DiDomenico GE, Njoroge WF, Barzilay R (2022). Association between discrimination stress and suicidality in preadolescent child. Journal of the American Academy of Child and Academy of Child and Adolescent Psychiatry.

[CR7] Barlow, A., & Okamoto, S. K. (2022). Dissemination and implementation of interventions to promote health equity with underserved populations. In F. G. Castro (Chairperson), *Strategic directions in preventive interventions to advance health equity.* Society for Prevention Research Annual Conference, Seattle, WA.

[CR8] Beets MW, Flay BR, Vuchinich S, Snyder F, Acock A, Burns K, Durlak J (2009). Use of social and character development program to prevent substance use, violent behaviors, and sexual activity among elementary-school students in Hawaii. American Journal of Public Health.

[CR9] Bender MS, Santos GM, Villanueva C, Arai S (2016). Development of a mobile phone-based weight loss lifestyle intervention for Filipino Americans with type 2 diabetes: Protocol and early results from the PilAm Go4Health randomized controlled trial. JMIR Research Protocols.

[CR10] Jernigan BB, V. D., Amico, E., Duran, B., & Buchwald, D.  (2020). Multilevel and community-level interventions with Native Americans: Challenges and opportunities. Prevention Science.

[CR11] Borowsky IW, Ireland M, Blum RW, Resnik MD (1998). Suicide attempts among American Indian-Alaska native youth: Risk and protective factors. Journal of Adolescent Health.

[CR12] Botvin GJ, Griffin KW, Diaz T, Ifill-Williams M (2001). Drug abuse prevention among minority adolescents: Posttest and one-year follow-up of a school-based preventive intervention. Prevention Science.

[CR13] Boyas JF, Kim YJ, Sharpe TL, Moore DJ, Prince-Stehley K (2017). An ecological path model of use of violence among African American adolescents. Child & Youth Services.

[CR14] Braveman P (2006). Health dispasrities and health equity: Concepts and measurement. Annual Review of Public Health.

[CR15] Braveman, P., Arkin, E., Orleans, T., Proctor, D., & Plough, A. (2017). *What is Health Equity? And what difference does a definition make?* R. W. J. Foundation.

[CR16] Brody GH, Chen YF, Kogan SM, Murry VM, Brown AC (2010). Long-term effects of the strong African American families program on youths’ alcohol use. Journal of Consulting and Clinical Psychology.

[CR17] Brody GH, Chen YF, Kogan SM, Yu T, Molgaard VK, DiClemente RJ, Wingood GM (2012). Family-centered program deters substance use, conduct problems, and depressive symptoms in black adolescents. Pediatrics.

[CR18] Bronfenbrenner, U. (1979). *The ecology of human development: Experiments by nature and design*. Harvard University Press.

[CR19] Brown BD, Harris KJ, Harris JL, Parker M, Ricci C, Noonan C (2010). Translating the diabetes prevention program for Northern Plains Indian youth through community-based participatory research methods. The Diabetes Educator.

[CR20] Brownson RC, Kumanyika SK, Kreuter MW, Haire-Joshu D (2021). Implementation science should give higher priority to health equity. Implementation Science.

[CR21] Bullinger LR (2017). The effect of minimum wages on adolescent fertility: A nationwide analysis. American Journal of Public Health.

[CR22] Burnet DL, Plaut AJ, Wolf SA, Huo D, Solomon MC, Dekayie G, Quinn MT, Lipton R, Chin MH (2011). Reach-out: A family-based diabetes prevention program for African American youth. Journal of the National Medical Association.

[CR23] Byrd TP, Wilson KM, Fernandez ME (2012). Using intervention mapping as a participatory strategy: Development of a cervical cancer screening intervention for Hispanic women. Health Education & Behavior.

[CR24] Cabassa, L. J., & Baumann, A. A. (2013). A two-way street: Bridging implementation science and cultural adaptations of mental health treatments. *Implementation Science*, Article 90. 10.1186/1748-5908-8-90 PMC3765289 23958445

[CR25] Castro FG, Hernandez-Alarcon E (2002). Integrating cultural variables into drug abuse prevention and treatment with racial/ethnic minorities. Journal of Drug Issues.

[CR26] Castro, F. G., & Kessler, R. (2021). Cultural factors in prevention. In W. O'Donohue & M. Zimmerman (Eds.), *Prevention of behavioral disorders in integrated care*. Springer Nature. 10.1007/978-3-030-83469-2_4

[CR27] Castro FG, Shaibi GQ, Boehm-Smith E (2009). Ecodevelopmental contexts for preventing type 2 diabetes in Latino and other racial/ethnic minority populations. Journal of Behavioral Medicine.

[CR28] Castro FG, Yasui M (2017). Advances in EBI development for diverse populations: Towards a science of implementation adaptation. Prevention Science.

[CR29] Centers for Disease Control & Prevention (CDC). (2020). *National Diabetes Statistics Report 2020: Estimates of diabetes and its burdens in the United States*. Atlanta, GA: U.S. Department of Health and Human Services

[CR30] Centers for Disease Control & Prevention (CDC). (2021). *Health disparities*. Retrieved 9–6–2021 from cdc.gov/aging/disparities/index.htm

[CR31] Charania MR, Crepaz N, Guenther-Gray C, Henny K, Liau A, Willis LA, Lyles CM (2011). Efficacy of structural-level condom distribution interventions: A meta-analysis of US and international studies, 1998–2007. AIDS and Behavior.

[CR32] Coatsworth JD, Pantin H, Szapocznik J (2002). Familias Unidas: A family-centered ecodevelopmental intervention to reduce risk for problem behavior among Hispanic adolescents. Clinical Child and Family Psychology Review.

[CR33] Coie JD, Watt NF, West SG, Hawkins JD, Asarnow JR, Marmkman HJ, Long B (1993). The science of prevention: A conceptual framework and some directions for a national research program. American Psychologist.

[CR34] Collins PH (2015). Intersectionality definitional dilemmas. Annual Review of Sociology.

[CR35] Cooper, D. G., & Christens, B. D. (2019). Justice system reform for health equity: A mixed methods examination of collaborating for equity and justice principles in a grassroots organizing coalition. *Health Education & Behavior*, *46*(IS), 625–705. 10.1177/1090198119859411 10.1177/1090198119859411 31549558

[CR36] Cox C (2020). Older adults and Covid 119: Social justice, disparities, and social work practice. Journal of Gerontological Social Work.

[CR37] Cresnshaw, K. (1989). Demarginalizing the intersection of race and sex: Black feminist critique of antidiscrimination doctrine, feminist theory and antiracist politics University of Chicago Legal Forum 139–168 10.4324/9780429500480-5

[CR38] Creswell, J. W., & Creswell, J. D. (2018). *Research design: Qualitative, quantitative, and mixed methods approaches* (5th ed.). Sage.

[CR39] Cwik MF, Tingey L, Maschino A, Goklish N, Larzelere-Hinton F, Walkup J, Barlow A (2016). Decreases in suicide deaths and attempts linked to the white mountain apache suicide surveillance and prevention system, 2001–2012. American Journal of Public Health.

[CR40] Damschroder, L. J., Aron, D. C., Keith, R. E., Kisch, S. R., Alexamder, J. A., & Lawry, J. C. (2009). Fostering implementation of health services research findings into practice: A consolidated framework for advancing implementation science. *Implementation Science*, *4*, Article 50. 10.1186/1748-5908-4-50 PMC2736161 19664226

[CR41] Dankwa-Mullan J, Perez-Stable EJ, Gardner KL, Zhang X (2021). The science of health disparities research.

[CR42] Diabetes Prevention Program Research Group (2002). The Diabetes Prevention Program (DPP): Description of lifestyle intervention. Diabetes Care.

[CR43] Dunkel Schetter C, Schafer P, Lanzi RG, Clark-Kauffman E, Raju TNK, Hillemeier MM (2013). Shedding light on the mechanisms underlying health disparities through community participatory methods: The stress pathway. Perspectives in Psychological Science.

[CR44] Fagan AA, Bumbarger BK, Barth RP, Bradshaw CP, Cooper BR, Supplee LH, Walker DK (2019). Scaling up evidence-based interventions in US public systems to prevent behavior health problems: Challenges and opportunities. Prevention Science.

[CR45] Fish J, Syed M (2018). Native Americans in higher education: An ecological systems perspective. Journal of College Student Development.

[CR46] Fishbein D (2021). The pivotal role of prevention science in this syndemic. Prevention Science.

[CR47] Ford CL, Airhihenbuwa CO (2010). Critical race theory, race equity, and public health: Towards antiracism praxis. American Journal of Public Health.

[CR48] Glasgow, R. E., Harden, S. M., Gaglo, B., Rabin, B., Smith, M. L., Porter, G. C., Ory, M. G., & Estabrooks, P. A. (2019). RE_AIM planning and evaluation framework: Adapting to new science and practice with a 20-year review. *Frontiers in Public Health*, *7*, Article 64. 10.3389/fpubh.2019.00064 PMC6450067 30984733

[CR49] Gottfredson DC, Cook TD, Gardner FE, Gorman-Smith D, Howe GW, Sandler IN, Zafft KM (2015). Standards of evidence for efficacy, effectiveness, and ccale-up research in prevention science: Next generation. Prevention Science.

[CR50] Green AE, Fettes DL, Aarons GA (2012). A Concept Mapping Approach to Guide and Understand Dissemination and Implementation Journal of Behavior Health Services & Research.

[CR51] Groos M, Wallace M, Hardeman R, Theall KP (2018). Measuring inequity: A systematic review of methods used to quantify structural racism. Journal of Health Disparities Research and Practice.

[CR52] Guo, Y., Lee, J., Rousseau, J., Pimentel, P., Bojorquez, Y., Cabasag, C., Silva, M., & Olshansky, E. (2016). The potential economic impact of a coordinated home visitation program: Preventing adverse birth outcomes. *California Journal of Health Promotion*, *14*(2), 1–13. 10.32398/cjhp.v14i2.1870

[CR53] Haroz EE, Ingalls A, Wadlin J, Kee C, Begay M, Neault N, Barlow A (2020). Utilizing broad-based partnerships to design a precision approach to implementing evidence-based home visiting. Journal of Community Psychology.

[CR54] Hawkins, J. D., Jenson, J. M., Catalano, R., Fraser, M. W., Botvin, G. J., Shapiro, V., Brown, C. H., Beardslee, W., Brent, D., Leslie, L. K., Rotheram-Borus, M. J., Shea, P., Shih, A., Anthony, E., Haggerty, K. P., Bender, K., Gorman-Smith, D., Casey, E., & Stone, S. (2015). *Unleashing the power of prevention*. Institute of Medicine and National Research Council.

[CR55] Hecht, M. L., Marsiglia, F. F., Elek, E., Wagstaff, D. A., Kulis, S., Dustman, P., & Miller-Day, M. (2003). Culturally grounded substance use prevention: An evaluation of the keepin' it R.E.A.L. curriculum. *Prevention Science*, *4*(4), 233–248. 10.1023/a:1026016131401 10.1023/a:1026016131401 14598996

[CR56] Israel, B. A., Shulz, A. J., Parker, E. A., Becker, A. B., Allen, A. J., Guzman, J. R., & Litchtenstein, R. (2018). Critical issues in developing and following CBPR principles. In N. Wallerstein, B. Duran, J. Oetzel, & M. Minkler (Eds.), *Community-based participatory research for health: Advancing social and health equity*. Jossey-Bass.

[CR57] Kegler, M. C., Wolff, T., Christens, B. D., Butterfoss, F. D., Francisco, V. T., & Orleans, T. (2019). Strengthening our collaborative approaches for advancing equity and justice. *Health Education & Behavior*, *46*(1_suppl), 5s-8s. 10.1177/1090198119871887 10.1177/1090198119871887 31549552

[CR58] Kellam SG, Langevin DJ (2003). A framework for understanding “evidence” in prevention research and programs. Prevention Science.

[CR59] Kenney A, Chambers RA, Rosenstock S, Neault N, Richards J, Reid R, Nelson L, Begay M, Grass R, Parker S, Barlow A (2016). The impact of a home-based diabetes prevention and management program on high-risk American Indian youth. The Diabetes Educator.

[CR60] Khare MM, Huber R, Carpenter RA, Balmer PW, Bates NJ, Nolen KN, Hudson HK, Lattyak RM, Cursio JF, Loo RK, Farris RP, Will JC (2009). A lifestyle approach to reducing cardiovascular risk factors in underserved women: Design and methods of the Illinois WISEWOMAN Program. Journal of Women's Health.

[CR61] Kneipp SM, Schwartz TA, Drevdahi DJ, Canales MK, Santacroce S, Santos HP, Anderson R (2018). Trends in health disparities, health inequity, and social determinants of health research. Nursing Research.

[CR62] Knowler WC, Barrett-Connor E, Fowler SE, Hamman RF, Lachin JM, Walker EA, Nathan DM (2002). Reduction in the incidence of type 2 diabetes with lifestyle intervention or metformin. New England Journal of Medicine.

[CR63] Koh HK, Oppenheimer SC, Massin-Short SB, Emmons KM, Geller AC, Viswanath K (2010). Translating research evidence into practice to reduce health disparities: A social determinants approach. American Journal of Public Health.

[CR64] Koh HK, Piotrowski JJ, Kumanyika S, Fielding JE (2011). Healthy people: A 2020 vision for the social determinants of health. Health Education & Behavior.

[CR65] Kok G, Gottlieb NH, Peters GJ, Mullen PD, Parcel GS, Ruiter RA, Fernández ME, Markham C, Bartholomew LK (2015). A taxonomy of behaviour change methods: An intervention mapping approach. Health Psychology Review.

[CR66] Krieger N (2012). Methods for scientific study of discrimination and health: An ecosocial approach. American Journal of Public Health.

[CR67] Kulis S, Dustman PA, Brown EF, Martinez M (2013). Expanding urban American Indian youths’ repertoire of drug resistance skills: Pilot results from a culturally adapted prevention program. American Indian and Alaska Native Mental Health Research.

[CR68] Kumanyika S (2019). Overcomming inequities in obesity: What don’t we know that we need to know?. Health Education & Behavior.

[CR69] Lee JP, Ponicki W, Mair C, Gruenewald P, Ghanem L (2020). What explains the concentration of off-premise alcohol outlets in black neighborhoods?. SSM Population Health.

[CR70] Leviton LC (2017). Generalizing about public health interventions: A mixed-methods approach to external validity. Annual Review of Public Health.

[CR71] Lightfoot, M. A., & Milburn, N. G. (2009). HIV prevention with African American youth: Examination of individual-level behaviour is not the only answer. Health & Sexuality, 11(7), 731–742 10.1080.13691050903078824 10.1080/13691050903078824 19657803

[CR72] Manson SM (2020). The role of culture in effective intervention design, implementation, and research: Its universal importance. Prevention Science.

[CR73] Martin CG, Fisher PA, Kim HK (2012). Risk for maternal harsh parenting in high-risk families from birth to age three: Does ethnicity matter?. Prevention Science.

[CR74] McCurley JL, Fortmann AL, Gutierrez AP, Gonzalez P, Euyoque J, Clark T, Preciado J, Ahmad A, Philis-Tsimikas A, Gallo LC (2017). Pilot test of a culturally appropriate diabetes prevention intervention for at-risk Latina women. The Diabetes Educator.

[CR75] Mena, E., Bolte G. (2019). Intersectionality-based quantitative health research and sex/gender sensitivity: *A scoping review Journal of Equity in Health, 18*. 10.1186/s12939-019-1098-8 10.1186/s12939-019-1098-8 PMC6925460 31864366

[CR76] Meyer IH (2003). Prejudice, social stress, and mental health in lesbian, gay, and bisexual populations: Conceptual issues and research evidence. Psychological Bulletin.

[CR77] Minkler, M., Rebanal, R. D., Pearce, R., & Acosta, M. (2019). Growing equity and health equity in perilous times: Lessons from community organizers. *Health Education & Behavior*, *46*(1_suppl), 9s-18s. 10.1177/1090198119852995 10.1177/1090198119852995 31549555

[CR78] Myers HF (2009). Ethnicity- and socio-economic status-related stresses in context: An integrative review and conceptual model. Journal of Behavioral Medicine.

[CR79] Nilsen P (2015). Making sense of implementation theories, models and frameworks. Implementation Science.

[CR80] Obasogie OK, Headen I, Mujahid MS (2017). Race, law, and health disparities: Toward a critical race intervention. Annual Review of Law and Social Science.

[CR81] Office of the Surgeon General. (2016). *Facing addiction in America: The surgeon general’s report on alcohol, drugs, and health*. U.S. Department of Health and Human Services. Retrieved March 8 from https://www.surgeongeneral.gov/library/2016alcoholdrugshealth/index.html 28252892

[CR82] Okamoto, S. K., Helms, S., Pel, S., McCain, L., Hill, A. P., & Hayashide, J. K. P. (2014). Developing empirically based, culturally grounded drug prevention interventions for indigenous youth populations. *Journal of Behavioral Health Services & Research, 41*(11), 8–19. 10.1007/s11414-012-9304-0 10.1007/s11414-012-9304-0 PMC3595362 23188485

[CR83] Okamoto SK, Kulis S, Helm S, Lauricella M, Valdez JK (2016). An evaluation of the Ho’ouna Pono Curriculum: A pilot study of culturally grounded substance abuse prevention for rural Hawaiian youth. Journal of Health Care for the Poor Underserved.

[CR84] Okamoto SK, Kulis S, Marsiglia FF, Holleran Steiker L, k., & Dustman, P.  (2014). A continuum of approaches toward developing culturally focused prevention interventions: From adaptation to grounding. Journal of Primary Prevention.

[CR85] Okamoto SK, Kulis SS, Helm S, Chin SK, Hata J, Hata E, Lee A (2019). An efficacy trial of the Ho’ouna Pono drug prevention curriculum: An evaluation of a culturally grounded substance abuse prevention program in rural Hawai’i. Asian American Journal of Psychology.

[CR86] Ordway MR, Sadler LS, Holland ML, Slade A, Close N, Mayes LC (2018). A home visiting parenting program and child obesity: A randomized trial. Pediatrics.

[CR87] Orengo-Aguayo R, Stewart RW, Villalobos BT, Rodriguez JH, Dueweke AR, de Arrellano M, Young J (2020). Listen, don’t tell. Partnership and Adaptation Trauma-Focused Cognitive Behavioral Therapy in Low-Resourced Settings American Psychologist.

[CR88] Pantin H, Prado G, Lopez B, Huang S, Tapia MI, Schwartz SJ, Sabillon E, Brown CH, Branchini J (2009). A randomized controlled trial of Familias Unidas for Hispanic adolescents with behavior problems. Psychosomatic Medicine.

[CR89] Pantin H, Schwartz SJ, Sullivan S, Prado G, Szapocznik J (2004). Ecodevelopmental HIV prevention programs for Hispanic adolescents. American Journal of Orthopsychiatry.

[CR90] Parent MC, DeBlaere C, Moradi B (2013). Approaches to research on intersectionality: Perspectives on gender, LGBT, and racial ethnic identities. Sex Roles.

[CR91] Parker M, Wallerstein NB, Duran B, Magarati M, Burgess E, Sanchez-Youngman S, Koegel P (2020). Engage for equity: Development of community-based participatory research tools. Health Education & Behavior.

[CR92] Penman-Aguilar A, Talih M, Huang D, Moonesinghe R, Bouye K, Backles G (2015). Measurement of health disparities, social determinants of health to support advancement of health equity. Journal of Public Health Management and Practice.

[CR93] Perrino T, Pantin H, Prado G, Huang S, Brincks A, Howe G, Beardslee W, Sandler I, Brown CH (2014). Preventing internalizing symptoms among Hispanic adolescents: A synthesis across Familias Unidas trials. Prevention Science.

[CR94] Philip J, Ford T, Hanry D, Rasmus S, Allen J (2016). Relationship of social network to protective factors in suicide and alcohol use disorder intervention for rural Yup’ik Alaska native youth. Psychological Intervention.

[CR95] Powell, B. J., Waltz, T. J., Chinman, M. J., Damschroder, L. J., Smith, J. L., Matthieu, M. M., Proctor, E. K., & Kirchner, J. E. (2015). A refined compilation of implementation strategies: Results from the Expert Recommendations for Implementing Change (ERIC) project Implementation Science 10. 10.1186/s13012-015-0209-1 10.1186/s13012-015-0209-1 PMC4328074 25889199

[CR96] Prado G, Cordova D, Huang S, Estrada Y, Rosen A, Bacio GA, Leon Jimenez G, Pantin H, Brown CH, Velazquez MR, Villamar J, Freitas D, Tapia MI, McCollister K (2012). The efficacy of Familias Unidas on drug and alcohol outcomes for Hispanic delinquent youth: Main effects and interaction effects by parental stress and social support. Drug and Alcohol Dependence.

[CR97] Price MA, Weisz JR, McKetta S, Hollinsaid NL, Lattanner MR, Reid AE, Hatzenbuehler ML (2022). Meta-analysis: Are psychotherapies less effective for Black youth in communities with high levels of anti-Black racism?. Journal of the American Academy of Child & Adolescent Psychiatry.

[CR98] Ramirez Garcia JI (2019). Integrating Latino/a determinants of health in research to promote population health and reduce health disparities. Cultural Diversity and Ethnic Minority Psychology.

[CR99] Resnicow K, Soler R, Braithwait RL, Ahluwaila JS, Butler J (2000). Cultural sensitivity in substance use prevention. Journal of Community Psychology.

[CR100] Rosenquist NA, Cook DM, Ehntholt A, Omaye A, Muenning P, Pabayo R (2020). Differential relationship between state-level minimum wage and infant mortality risk among US infants born to white and black mothers. Journal of Epidemiology and Community Health.

[CR101] Sallis, J. F., & Owen, N. (2015). Ecologica models of health behavaior. In K. Glanz, B. K. Rimer, & K. Viswanath (Eds.), *Health Behavior: Theory, Research, and Practice* (Fifth Edition ed., pp. 43–64). Jossey-Bass.

[CR102] Sauder KA, Dabelea D, Bailey-Callahan R, Kanott Lambert S, Powell J, James R, Percy C, Jenks BF, Testaverde L, Thomas JM, Barber R, Smiley J, Hockett CW, Zhong VW, Letourneau L, Moore K, Delamater AM, Mayer-Davis E (2018). Targeting risk factors for type 2 diabetes in American Indian youth: The Tribal Turning Point pilot study. Pediatric Obesity.

[CR103] Sheftall AH, Boyd RC, Ackerman JP, Horowitz LM (2022). Black youth suicidal behavior: What we know and where we go from here. Youth Suicide Prevention and Intervention: Best Practices and Policy Implications.

[CR104] Shelton, R. C., & Adsul, P. (2021). Recommendations for addressing structural racism in implementation science: A call to the field. *Ethnicity and Disease*, 357–364. 10.18865/ed.31.S1.357 10.18865/ed.31.S1.357 PMC8143847 34045837

[CR105] Skivington, K., Matthews, L., Simpson, S. A., Craig, P., Baird, J., Blazeby, J. M., Boyd, K. A., Craig, N., French, D. P., McIntosh, E., Pettigrew, M., Rycroft-Malone, J., White, M., & Moore, L. (2021). A new framework for developing and evaluating complex interventions: Update of medical research council guidance *British Medical Journal*, *374*. 10.1136/bmj.n2061 10.1136/bmj.n2061 PMC8482308 34593508

[CR106] Smith JD (2022). The Implementation Research Logic Model (IRLM): A method for planning and executing implementation research projects.

[CR107] Smith JD, Li DH, Rafferty MR (2020). The implementation research logic model: A method for planning, executing, reporting, and synthesizing implementation projects. Implementation Science.

[CR108] Sorkin DH, Mavandadi S, Rook KS, Biegler KA, Kilgore D, Dow E, Ngo-Metzger Q (2014). Dyadic collaboration in shared health behavior change: The effects of a randomized trial to test a lifestyle intervention for high-risk Latinas. Health Psychology.

[CR109] Sterling, M. R., Echeverria, S. E., Commodore-Mensah, Y., Breland, J. X., & Nunez-Smith, M. (2019). Health equity and implementation science in heart, lung, blood, and sleep-related research: Emerging themes from the 2018 Saunders-Watkins leadership workshop. *Circulation: Cardiovascular Quality and Outcomes*. 10.1161/CIRCOUTCOMES.119.005586. 10.1161/CIRCOUTCOMES.119.005586 PMC6812546 31610713

[CR110] Sun S, Hoyt WT, Tarantino N, Pachankis JE, Whiteley L, Oparario D, Brown LK, &,  (2020). Cultural context matters: Testing the minority stress model among Chinese sexual minority men. Journal of Counseling Psychology.

[CR111] Taket, A., & McKay, F. H. (2022). Health equity and human rights. In F. H. McKay & A. Taket (Eds.), *Health equity, social justice and human rights*. Taylor & Francis.

[CR112] Tapager I, Olsen KR, Vrangbaek K (2022). Exploring equity in accessing diabetes management treatment: A health care gap analysis. Social Science & Medicine.

[CR113] Thomson JL, Tussing-Humphreys LM, Goodman MH, Landry AS (2018). Enhanced curriculum intervention did not result in increased postnatal physical activity in rural, southern, primarily African American women. American Journal of Health Promotion.

[CR114] Trinh-Shevrin, C., S., N., Park, R., Islam, N., & Kwon, S. C. (2015). Defining an integrative approach for health promotion and disease prevention: A population health equity framework *Journal of Health Care for the Poor Underserved*, *26*(2 Suppl), 146–163. 10.1353/hpu.2015.0067 10.1353/hpu.2015.0067 PMC4530990 25981095

[CR115] U. S. Department of Health & Human Services. (2019). *Healthy People 2020: Disparities*. Retrieved March 8 from https://www.healthypeople.gov/2020/about/foundation-health-measures/Disparities

[CR116] Van Ryzing M, Fishbein D, Biglan A (2018). The promise of prevention science for addressing intergenerational poverty. Psychology, Public Policy, & Law.

[CR117] Volpe VV, Dawson DN, Rahal D, Wiley KC, Vesslee S (2019). Bringing psychological science to bear on racial health disparities: The promise of centering Black health through a critical race framework. Translational Issues in Psychological Science.

[CR118] Votruba ME, Kling JT (2009). Effects of neighborhood characteristics on the mortality of black male youth: Evidence from Gautreaux. Chicago. Social Science & Medicine.

[CR119] Walker RJ, Smalls BL, Campbell JA, Strom Williams JL, Egede LE (2014). Impact of social determinants of health on outcomes for type 2 diabetes: A systematic review. Endocrine.

[CR120] Wallerstein, N., Duran, B., Oetzel, J., & Minkler, M. (2018). *Community-based participatory research for health: Advancing social and health equity*. Jossey-Bass.

[CR121] Wallerstein NB, Duran B (2006). Using community-based participatory research to address health disparities. Health Promotion Practice.

[CR122] Woodward EN, Matthieu MM, Uchendu US, Rogal S, Kirchner JE (2019). The health equity implementation framework: Proposal and preliminary study of hepatitis C virus treatment. Implementation Science.

